# Statistical shape modeling of MRI-based morphological response of lumbar intervertebral discs to a unilateral side lying spinal rotation mobilization

**DOI:** 10.1016/j.ocarto.2026.100783

**Published:** 2026-03-14

**Authors:** Joseph Putos, Hayden F. Atkinson, David M. Walton

**Affiliations:** a School of Physical Therapy, Western University, London, Canada; b Department of Orthopaedics, London Health Sciences Centre, London, Canada; c Western Bone & Joint Institute, Western University, London, Canada

**Keywords:** Intervertebral disc, Magnetic resonance imaging, Statistical shape modeling, Spinal mobilization, Low back pain

## Abstract

**Objectives:**

The acute response of lumbar intervertebral discs (IVD) to spinal rotation mobilization (SRM) is poorly understood. We evaluated sustained morphological changes in lumbar IVDs following a rotation mobilization commonly used to relieve discogenic pain.

**Design:**

In this cross-sectional study, five asymptomatic participants underwent MRI of the lumbar spine before and after 2 min of sustained passive unilateral SRM. Pre-SRM acquisition was completed twice, repositioning between acquisitions to assess test-retest reliability. Five lumbar IVDs per participant were segmented independently by two raters, and compared between timepoints using Dice similarity coefficients (DSC). We used statistical shape modeling (SSM) to assess magnitude and direction of shape changes as defined by principal component analysis (PCA). Primary analysis evaluated the PCA scores from the SSM models of all IVDs; secondary analysis assessed each IVD individually.

**Results:**

Significant changes were observed in IVD shape following SRM (p < 0.001), represented by significant differences in PCA scores following SRM for two modes (Mode 4 PCA score difference: −2.60 [95%CI: −4.79; −0.41]; Mode 7: −1.51 ([−2.65; −0.37]), demonstrating anterolateral IVD expansion ipsilateral to rotation direction. Similar results were observed in the secondary analyses for mode 3 of the L1-L2 IVD (−6.28 [−11.03; −1.51], p = 0.01) and mode 4 of the L2-L3 IVD (−4.08 [−8.13; −0.04], p = 0.02), with no significant changes in more caudal IVDs. Reliability within and between raters and timepoints was excellent (mean DSC >0.9).

**Conclusions:**

SRM induces measurable, direction-specific changes in healthy IVDs. Future research should evaluate symptomatic IVDs, examining relationships between morphology, symptoms, and therapeutic parameters.

## 1 Introduction

Low back pain (LBP) is a common musculoskeletal condition, with nearly 80% of the global population experiencing one or more episodes in their lifetime [[1], [2], [3]]. As a result, LBP is the leading cause of years lived with disability (YLDs), representing a high patient and economic burden [4]. While many cases of LBP are classed as ‘non-specific’, discogenic (dLBP) is among the more common patho-anatomically identifiable forms of LBP. dLBP is often associated with an acute onset and predictable pattern of mechanically influenced aggravation and relief of symptoms with or without radiation to the lower extremity [1,5]. Symptoms typically resolve over time as part of the natural history of the condition, often supported by therapeutic symptom modification techniques, including exercise, manual therapy, and education, as well as pharmaceutical and passive modalities when indicated [6,7]. In some cases, non-resolving discogenic LBP is treated operatively by removing gross abnormalities such as IVD protrusions or relieving pressure on surrounding spinal structures, with varying degrees of success [1,6]. Surgical approaches, such as microdiscectomy or decompression, are options for patients with persistent symptoms though outcomes vary depending on patient characteristics and the severity of the underlying pathology [8,9]. Given the challenge of assessing the behavior of IVD in vivo, the relationship between IVD shape, function, symptoms, and response to intervention is poorly understood.

Patients with discogenic LBP commonly exhibit symptom relief in specific directions of spinal movement, and concurrently experience symptom aggravation in generally opposite directions, suggesting a relationship between spinal posture and mechanical load on the IVD [[10], [11], [12], [13], [14]]. Clinically this presents as a predictable, repeatable nociceptive response [5,13,15]. This phenomenon is often attributed to changes in intradiscal pressure and mechanical stress on nociceptive structures, such as the annulus fibrosus and surrounding ligaments. However, given the challenge of simultaneously measuring real-time symptoms in accordance with the physiological behavior of the IVD and surrounding spinal structures, direct mechanistic relationships have yet to be established for the explanation of acute changes in discogenic LBP symptoms. One persistent theory proposes that pain in at least a subset of people can be attributed to IVD herniations compressing adjacent spinal tissues, such as the posterior longitudinal ligament (PLL) and ventral dura mater, which are innervated with mechano- and chemo-sensitive nociceptors [5,16,17]. This theory suggests that interventions such as spinal mobilization can normalize the position of the IVD, thereby relieving pain and improving function [18,19]. While acknowledged as a clinical entity, there remains limited research to confirm or refute these mechanisms. Hypotheses to explain acute symptom changes have included combinations of mechanical, neuropathic, central sensitization, behavioral activation, and placebo effects. Better understanding IVD behavior is essential for the study, prevention, and management of dLBP.

Previous research has evaluated changes in the position and orientation of the surrounding lumbar vertebrae using computed tomography (CT) following a spinal rotational mobilization and thereby inferred the shape of the IVD from these findings. Xu et al. [13] investigated the effects of axial torsion on IVD height distribution in vivo using CT imaging in 81 participants, assessing changes in IVD morphology during supine and rotated positions. The study used virtual models of the intervertebral gaps to analyze five anatomical zones corresponding to regions of the annulus fibrosus and nucleus pulposus. They observed significant changes in IVD height distribution, particularly in the peripheral zones, indicating that rotational mobilization influences IVD morphology. While this study provides some interpretation of the change in IVD shape in response to loading, the use of CT and inference of IVD shape from neighboring bony structures is not a fully representative model of the non-rigid nature of the IVD, missing crucial information on changes in shape around the peripheral radius of the IVD not represented by the bony surface of the vertebral endplates. Moreover, most previous studies have relied on 2D analyses of IVD shape, which fail to capture the complex 3D morphology of IVDs [20,21]. This limitation highlights the need for advanced imaging techniques capable of providing detailed 3D assessments of IVD behavior.

The purpose of this study was to evaluate the change in IVD shape following sustained positioning of spinal rotation in a side-lying position that is commonly used for the treatment technique of passive therapeutic spinal rotation mobilization (SRM). We used high resolution magnetic resonance imaging (MRI) and statistical shape modeling (SSM) to model alterations in IVD shape, providing soft tissue contrast and robust methodology to evaluate IVD behavior. The rationale for the design is that this mobilization, commonly used by manual therapists, is theorized amongst practitioners to affect the shape of the IVD thereby relieving mechanical pressure on local nociceptor-innervated tissues in the presence of herniation. While some variation in the application of the mobilization technique exists, consistent is that the patient is in a side-lying position while the clinician/therapist applies rotational force directed through the affected vertebral level(s) to affect shape or position of the IVD. We hypothesize that IVD shape will change following the sustained side-lying spinal rotation position, specifically in the anterolateral direction, ipsilateral to the direction of rotation, resulting in redistribution of IVD matter away from the opposite posterolateral side of the IVD. We also hypothesized no change in IVD volume.

## 2 Methods

### 2.1 Participants

Using a cross-sectional, repeated-measures design, a convenience sample of five participants with no history of low back pain or sciatica for the last six months and no contraindications to MRI scanning, were recruited from a group of students at Western University between January 2017 and February 2019. Participants provided written informed consent to participate in the study, approved by the Western University Research Ethics Board before undergoing study procedures. Given the exploratory nature of the study, we did not perform a sample size calculation.

### 2.2 Imaging protocol

Upon arrival, participants completed a numeric pain rating scale for LBP and underwent a standardized assessment of active lumbar range of motion by an experienced physical therapist to ensure no current spinal impairments. Participants were then seated comfortably for 20 min while completing study-specific demographic information. The seated time as well as the standardized start time of the protocol (between 14:00 and 16:00 for all participants) was intended to normalize spinal loading prior to completing the scan to account for the effects of accumulated load throughout the day [22].

Following the 20-min load standardization, participants underwent three separate acquisition timepoints (A1, A2, A3) within 50 min in a 3.0 T S Magnetom Trio scanner using a surface spine coil. All three acquisitions included high-resolution near-isotropic (0.94 × 0.97 × 0.90 mm) sagittal T1 volumetric interpolated breath-hold examination (VIBE) and isotropic (0.94 × 0.97 × 0.90 mm) T2 sampling perfection with application optimized contrasts with flip angle evolutions (SPACE), both 3D sequences. Sequence parameters are presented in Table 1. Following image acquisition from Participant 1, it was determined that the T1 VIBE sequence provided superior contrast at the disc-bone interface. We maintained the same imaging protocol for the remainder of the participants to ensure consistency in the time between A1, A2, and A3.

**Table 1:** MRI sequences.

Sequence	TR (ms)	TE (ms)	FOV (mm)	Matrix Size (pixels)	Slice Width (mm)	Resolution (mm)	Slice Gap (mm)	Scan time (mins)
T1 VIBE Dixon	5.8	2.46, 3.69	300 × 282	320 × 290	0.90	0.94 × 0.97 × 0.90	0.2	10:46
Sagittal								
T2 3D SPACE	1500	144	300 × 300	320 × 320	0.94	0.94 × 0.94 × 0.94	–	5:57
Sagittal								

TR = repetition time, TE = echo time, TSE = turbo spin echo, FOV = field of view, ms = milliseconds, VIBE = volumetric interpolated breath-hold examination, SPACE = sampling perfection with application optimized contrasts with flip angle evolutions.

Both sequences were repeated a total of three times, twice for the baseline/reliability measures, and a third time following the rotation. The T1 VIBE sequence was used for segmentation purposes.

**Table 2:** Measured lumbar IVD volume (in mm^3^) at each timepoint for each participant.

	Participant					Mean ± SD/MD (95%CI)	p
	1	2	3	4	5		
L1-L2 A1	9,898.0	10,347.8	7,157.6	8,903.4	21,592.8	11,579.9 ± 5,729.9	
L1-L2 A2	10,700.4	10,581.6	6,520.2	9,480.9	19,558.1	11,368.2 ± 4,878.6	
L1-L2 A12 Δ	802.4	233.8	−637.4	577.5	−2,034.7	−211.7 (−1,225.8; 802.4)	0.70
L1-L2 A3	9,995.2	12,074.9	6,474.0	9,253.8	24,445.6	12,448.7 ± 6,999.8	
L1-L2 SRM Δ	−304.0	1,610.2	−364.9	61.6	3,870.2	974.6 (−608.7; 2,557.9)	0.23
L2-L3 A1	12,987.7	11,504.2	10,193.4	9,997.8	22,328.5	13,402.3 ± 5,131.7	
L2-L3 A2	14,156.5	11,504.5	11,057.9	10,123.5	20,348.6	13,438.2 ± 4,143.1	
L2-L3 A12 Δ	1,168.8	0.3	864.5	125.7	−1,979.9	35.9 (−1,041.5; 1,113.2)	0.95
L2-L3 A3	13,329.5	12,741.0	9,820.5	10,730.6	22,174.8	13,759.3 ± 4,917.6	
L2-L3 SRM Δ	−242.6	1,236.7	−805.2	670.0	836.3	339.0 (−395.64; 1,073.7)	0.37
L3-L4 A1	14,184.4	14,277.5	13,472.0	12,264.2	22,187.4	15,277.1 ± 3,945.9	
L3-L4 A2	14,554.8	15,120.1	12,648.6	11,985.4	21,319.8	15,125.7 ± 3,697.6	
L3-L4 A12 Δ	370.4	842.6	−823.4	−278.8	−867.6	−151.4 (−807.5; 504.7)	0.66
L3-L4 A3	13,573.5	14,856.4	12,456.5	12,936.7	22,575.3	15,279.7 ± 4,176.6	
L3-L4 SRM Δ	−796.1	157.6	−603.8	811.9	821.7	78.3 (−590.3; 746.8)	0.83
L4-L5 A1	12,470.2	14,925.2	13,370.2	12,409.3	24,288.8	15,492.7 ± 5,021.0	
L4-L5 A2	14,098.5	14,623.1	12,379.1	12,733.8	23,934.4	15,553.8 ± 4,776.2	
L4-L5 A12 Δ	1,628.3	−302.1	−991.1	324.5	−354.4	61.0 (−808.6; 930.7)	0.90
L4-L5 A3	12,467.7	15,109.1	13,268.2	12,821.9	24,637.1	15,660.8 ± 5,120.0	
L4-L5 SRM Δ	−816.7	334.9	393.5	250.4	525.5	137.5 (−338.2; 613.3)^W^ *137.5 (*−*338.2; 613.2)*	0.58 *0.58*
L5-S1 A1	9,869.1	14,291.6	13,959.1	9,656.5	17,080.4	12,971.3 ± 3,304.0	
L5-S1 A2	11,285.1	13,273.2	14,278.6	9,022.9	17,615.5	13,095.1 ± 2,820.3	
L5-S1 A12 Δ	1,416.0	−1,018.4	319.5	−633.6	535.1	123.7 (−725.6; 973.1)	0.79
L5-S1 A3	10,619.8	14,589.9	14,373.5	10,452.1	18,648.7	13,736.8 ± 3,345.9	
L5-S1 SRM Δ	42.7	807.5	254.6	1,112.4	1,300.8	**703.6 (29.0; 1,178.3)**	**0.02**

Group mean and standard deviation (SD), 95% confidence intervals (95%CI), and mean differences (MD) represent IVD volume, and any changes between pre- and post-spinal rotation manipulation (SRM) acquisitions. A12: For pre- and post-SRM comparisons, the pre-rotation volume was averaged between A1 and A2. Bolded results represent significant differences. A superscripted “W” (^W^) represents a positive Shapiro-Wilk test. Italicized results represent the adjusted values after permutation testing.

**Fig. 1:**
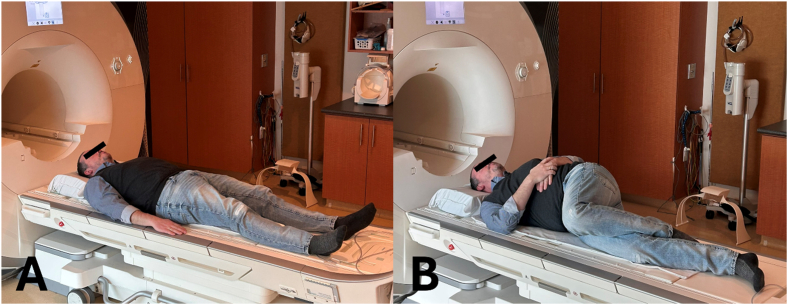
A) Standardized participant scanning positioning for scans at all 3 timepoints, supported under the head and knees with a pillow. The collapsible spine coil rests between the lumbar spine and the gurney. B) Depiction of the rotated position patients maintained for 2 min following timepoint A2 prior to returning to the supine position for scan A3.

The scans for A1 were collected immediately after the normalized sitting protocol. Participants were positioned in standard supine spinal acquisition positioning on the gurney, with the spine coil against their lower back (Fig. 1a). The A1 scan sequence took approximately 17 min to acquire the T1 VIBE and T2 SPACE sequences. Between A1 and A2 scans, the gurney was retracted from the scanner while the participant remained lying supine. The participant remained in the same position without moving and was observed by the technician. After 2 min, the participant re-entered the bore and the A2 scan was completed, repeating the same sequences. After A2, the table was retracted, and the participant was repositioned into a right side-lying position to emulate the SRM. The participants’ spine was rotated such that the left hemipelvis was rotated anteriorly (right spinal rotation of the inferior segment) and the torso was rotated posteriorly (left spinal rotation of the superior segment), similar to the position used during common SRM procedures (Fig. 1 a, b). As these were otherwise asymptomatic participants there was no indication for manual pressures to be applied, rather the participant remained in the comfortably rotated position for 2 min. After the 2-min hold, the participant was again repositioned into the supine-lying position, returned to the bore, and the acquisition was repeated for the post-SRM A3.

### 2.3 Image processing

#### 2.3.1 Segmentation

**Fig. 2:**
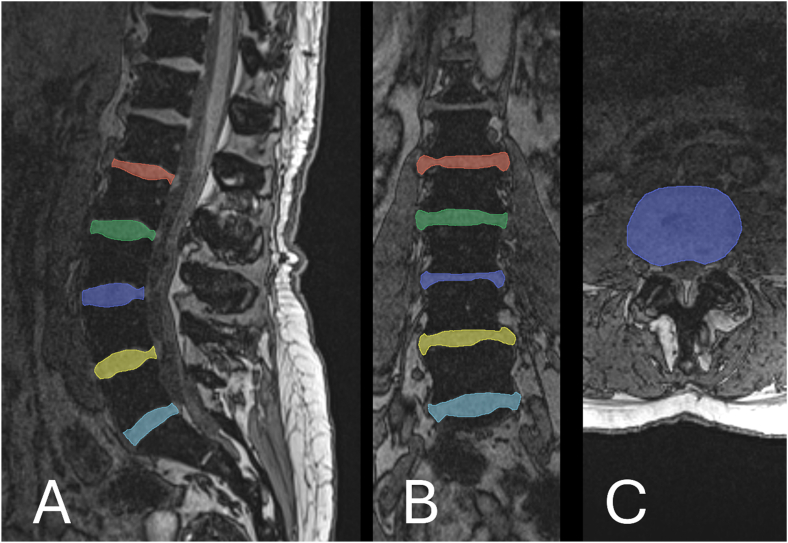
Example of the lumbar IVD segmentations in the sagittal (A), coronal (B), and axial (C) planes using the T1 VIBE sequence.

The T1 VIBE sequence was used to manually segment each of the 5 lumbar IVDs in all three planes using ITK Snap 3.6.0 [23]. Two raters segmented all images independently. The first rater (JP) was trained to use image processing software, taking advantage of 40 years of anatomical and clinical familiarity with spinal anatomy. The second rater (HFA), with 10 years of musculoskeletal image segmentation and processing experience, completed independent repeated segmentations to verify the reliability of the first rater's segmentations. Both raters were blinded to participant, acquisition timepoint, and their counterpart's segmentations. An example of a completed segmentation is demonstrated in Fig. 2.

#### 2.3.2 Statistical Shape Modeling

We used ShapeWorks Studio 6.5.1 [24] to preprocess and conduct SSM analyses. For the primary analysis, segmented images of each of five lumbar IVDs of all five participants at each timepoint were used to analyze overall changes in shape across all IVDs. To minimize segmentation-related variability, the first and second timepoints were merged into a single representative mesh using particle-based correspondence and compared to the third timepoint.

Batch preprocessing included a Laplacian smoothing filter with 100 iterations for the meshes of the primary analysis and 50 iterations for the secondary analyses. Each smoothed mesh was then represented using 256 equidistant surface particles to establish correspondences across all timepoints. We used a Generalized Procrustes Analysis to control for IVD size and align all IVDs in a common orientation and coordinate system. This controlled for differences in IVD size, translation, and rotation, as a statistical shape variable, allowing us to identify true changes in morphology as the primary outcome.

### 2.4 Statistical analyses

#### 2.4.1 Inter & intra-rater reliability

We assessed test-retest reliability of IVD segmentations using Dice similarity coefficients (DSC) and intraclass correlation coefficients (ICC) within and between raters. DSC is a measure of volumetric overlap between two segmentations of the same region of interest, and ICC reflects the agreement of the total volume values between raters. A DSC value of 1.0 indicates perfect overlap, while a value of 0 indicates no overlap. Test-retest reliability was assessed within raters by comparing segmentations at the two repeated baseline timepoints to account for segmentation and imaging consistency. Inter-rater reliability was evaluated by comparing segmentations between raters across all IVDs in the sample at each timepoint. Coppock et al. [25] have previously reported DSC values of 0.98 and ICC of 0.93 using a deep learning model trained on 25 subjects.

#### 2.4.2 Volumetric analysis

The total volume of each IVD (mm^3^), was extracted from each segmentation as a quantitative output using ITK Snap, and was compared at all three timepoints. The two baseline timepoints were compared for reliability, then mean volume of the two (A12) was used to represent the pre-rotation volume to reduce potential error in the remaining analyses attributable to patient positioning and segmentation inconsistencies. Dependent samples t-tests were conducted for each IVD to test for significant changes in volumes between A12 and A3. Acknowledging that each disc may respond differently to the protocol and in either direction, we treated all analyses as individual hypotheses using two-tailed tests. We conducted Shapiro-Wilk tests to assess normality of distribution. In the event of a violation, we performed paired permutation testing with 1000 random iterations to test our hypotheses in place of dependent samples t-tests, which provide robust inference in small samples without distributional assumptions [26].

#### 2.4.3 Statistical Shape Modeling

We used principal component analysis (PCA) to identify the top 10 PCs, explaining 81.5% and 96.5–98.2% (primary and secondary analyses, respectively) of the variance in IVD shape meshes following SRM. PCA was applied to quantify the primary modes of variation in shape between timepoints, ensuring that the analysis captured the most significant and apparent patterns of morphological change. For each of the top 10 PCs, we conducted paired t-tests to compare PC scores between the A12 and A3 timepoints. This approach assessed whether statistical shape differences represented by the PC scores were statistically significant from baseline. For our primary analysis, we included all IVDs from all participants and compared the shape changes between the pre- and post-SRM timepoints to assess the general response of all lumbar IVDs. For our secondary analysis, we took the same statistical approach, comparing each individual IVD segment across time to evaluate the unique responses of the L1-L2 through L5-S1 segments. The outcome measures for both the primary and secondary analyses were the mean differences and 95% confidence intervals for the principal component scores of the first 10 modes, based on ±2 SD of variation. As with the volumetric analysis, we assessed for normality of distribution and applied paired permutation testing in the event of a violation, and treated each analysis as independent hypotheses given that disc segments may respond differently to the protocol.

## 3 Results

A total of five participants with no recent history of LBP consented to participate (4 male, 1 female, age range 25–36, weight range 63.5–81.8 kg, height range 1.63–1.90 m, BMI range 22.7–27.2 kg/m^2^). All five completed all parts of the study with no dropouts or withdrawals.

### 3.1 Reliability

Intra-rater reliability was excellent for both rater 1 (mean DSC [95%CI]: 0.94 [0.89 to 0.99]) and rater 2 (0.95 [0.93 to 0.97]) across all timepoints. Inter-rater reliability was also excellent (0.90 [0.84 to 0.96]). These results demonstrate comparable reliability and agreement to the deep learning model developed by Coppock et al. [25].

### 3.2 Volumetric analysis

IVD volume remained consistent when comparing A1 vs A2 for all segments, with no significant differences observed between the two resting conditions. When comparing the pre-versus the post-SRM A3, there were no further differences in IVD volume with the exception of the L5-S1 segment, which demonstrated a small increase of 703.6 mm^3^ (95%CI: 29.0; 1178.3, p = 0.02), approximately 5% (95%CI: 0.2; 8.4%) of the original volume. Shapiro-Wilk test was significant for violation normality for the change in volume between the L4-L5 segment (W = 0.73, p = 0.02), however paired permutation testing confirmed the L4-L5 volume change was non-significant (Δ = 137.5 mm^3^ [−338.2; 613.2], p = 0.6). Volumetric analysis results are presented in Table 2.

### 3.3 Statistical shape modeling: primary analysis

**Fig. 3:**
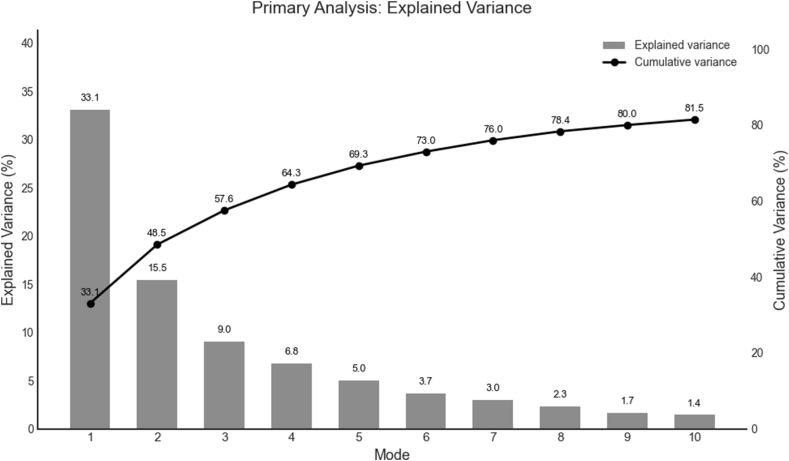
Scree plot showing the explained variance for each mode included in the primary analysis (represented in bars) and the cumulative variance explained across modes (black line).

**Fig. 4:**
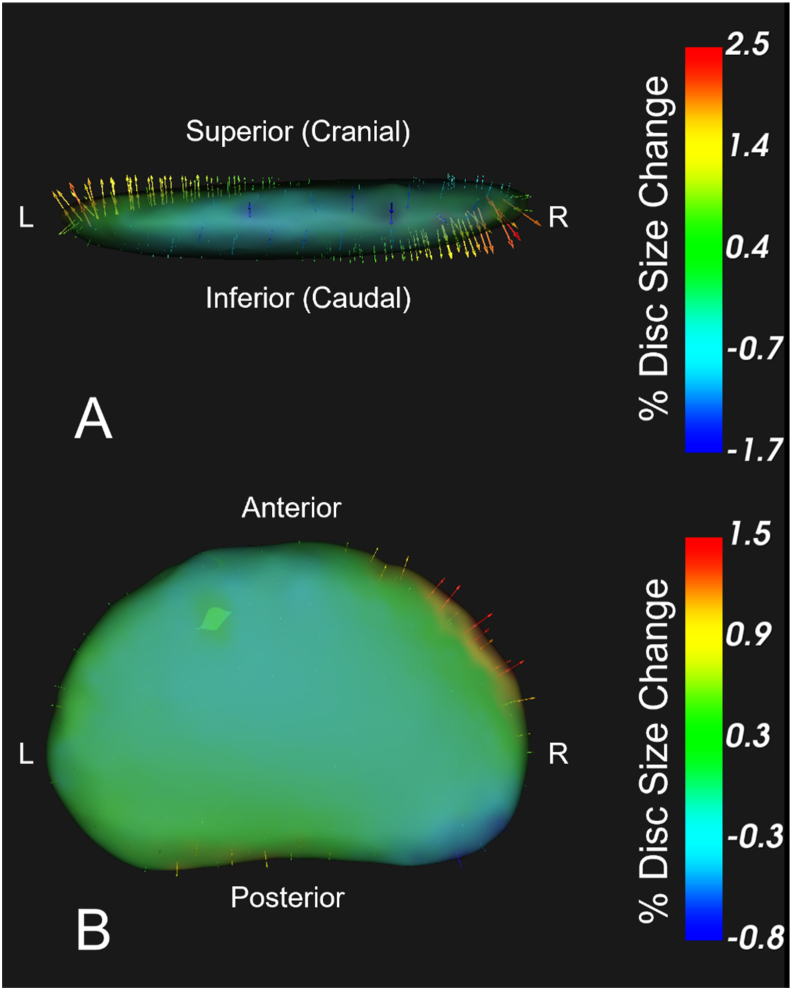
Visualization of modes 4 (Fig. 4A) and 7 (Fig. 4B) of the primary analysis, demonstrating significant changes in IVD shape following the rotation mobilization. Green to red vectors represent expansion and blue vectors represent regression. Units are represented in the percentage of the IVD size in the direction of the vector. 4A demonstrates expansion in the left superolateral and right inferolateral regions, consistent with coupled ipsilateral rotation and side bend expected with right rotation. 4B demonstrates right anterolateral expansion consistent and right posterolateral regression, consistent with coupled ipsilateral rotation and extension expected with right rotation. Range of disc shape change represents ±2 standard deviations.

PCA identified significant changes in IVD shape for two of the top 10 PCs in the primary analysis, which evaluated collective change in IVD shape in all segments to assess the general effect of the SRM on lumbar IVDs (p < 0.001). The top 10 modes explained 81.5% of the variance in shape change (Fig. 3). These significant principal components represented distinct and complementary changes in IVD shape. The first significant principal component (Mode 4, explained variance = 6.8%) demonstrated rotational changes in IVD shape (PC score difference: −2.60 [95%CI: −4.79; −0.41], p = 0.02). The representative shape deformation model is visualized in Fig. 4A (values summarized in Table 3), with this PC mode representing up to a 2.5% expansion in IVD size in the left superolateral and right inferolateral regions, and a −1.7% retraction of the left inferolateral and right superolateral regions.

**Table 3:** Participant and group PC scores for all significant changes in IVD shape in the primary and secondary analyses.

	Participant					Mean (95%CI MD)	p
	1	2	3	4	5		
Primary analysis (all IVDs)							p-value
**Mode 4 – A12**	−0.47	−6.33	−5.43	10.24	6.33	0.87 ± 6.50	
**Mode 4 – A3**	0.59	−5.76	−7.94	7.96	−3.52	−1.73 ± 5.61	
**Mode 4 – Δ**	1.06	0.57	−2.51	−2.28	−9.85	**−2.60 (−4.79; −0.41)**	**0.02**
**Mode 7 – A12**	−6.19	3.47	3.00	3.04	−0.80	0.50 ± 3.69	
**Mode 7 – A3**	−5.99	0.92	2.23	1.63	−3.82	−1.01 ± 3.28	
**Mode 7 – Δ**	0.20	−2.55	−0.77	−1.41	−3.02	**−1.51 (−2.65; −0.37)**	**0.01**

Secondary analysis (L1-L2)							
**Mode 3 – A12**	−8.81	−2.69	8.57	8.76	4.63	2.09 ± 6.85	
**Mode 3 – A3**	−13.29	−7.37	3.45	4.76	−8.47	−4.18 ± 7.07	
**Mode 3 – Δ**	−4.48	−4.68	−5.12	−4.00	−13.1	**−6.28 (−11.03; −1.51)^W^** ***−6.28 (*−*9.64;* −*2.92)***	**0.01** ***0.04***

Secondary analysis (L2-L3)							
**Mode 4 – A12**	−8.75	2.64	5.73	5.51	1.68	1.36 ± 5.30	
**Mode 4 – A3**	−10.24	1.00	2.89	0.34	−7.60	−2.72 ± 5.20	
**Mode 4 – Δ**	−1.49	−1.64	−2.84	−5.17	−9.28	**−4.08 (−8.13; −0.04)**	**0.047**

Group mean, 95% confidence intervals (95%CI), and mean differences (MD) presented are represented in principal component (PC) scores. PC scores represent each participant's position on a given shape axis for a particular mode before (Pre) and after (Post) the rotation mobilization. Shape changes are visualized in Fig. 3, Fig. 4. Bolded results represent significant differences. A superscripted “W” (^W^) represents a positive Shapiro-Wilk test. Italicized results represent the adjusted values after permutation testing.

Results for all modes of the primary analysis and all modes from the L1-L2 and L2-L3 secondary analyses are presented in Supplementary Tables 1, 2, and 3. AT12 = mean PC scores of acquisitions 1 and 2; AT3 = third (post SRM) acquisition, **Δ** = change, p = p-value.

The second significant principal component (Mode 7, explained variance 3.0%) primarily demonstrated peripheral changes in IVD shape (PC score difference: −1.51 ([−2.65; −0.37], p = 0.01). The representative shape deformation model is visualized in Fig. 4B (values summarized in Table 3), with this PC mode representing a right anterolateral expansion of up to 1.5% and a concurrent right posterolateral retraction of 0.8%.

### 3.4 Statistical shape modeling: secondary analysis

In analyses by IVD level, significant changes in shape were observed in the L1-L2 (Mode 3) (p = 0.02), and L2-L3 (Mode 4) IVDs (p = 0.01). These changes were consistent with those observed with the primary analysis, characterized by rotational differences in IVD shape. We did not identify any significant changes in shape in the remaining IVDs (L3-L4, L4-L5, L5-S1).

**Fig. 5:**
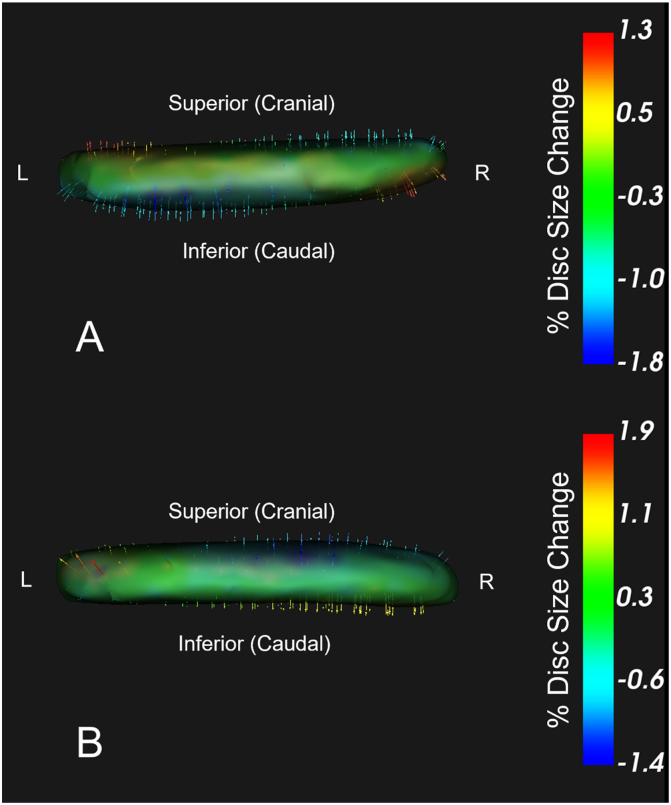
Visualization of mode 3 from the L1-L2 analysis (Fig. 5A) and mode 4 from the L2-L3 analysis (5B), demonstrating significant changes in IVD shape following the rotation mobilization, similar to the shape changes demonstrated in Fig. 5B. Green to red vectors represent expansion and blue vectors represent regression. Units are represented in the percentage of the IVD size in the direction of the vector. Both IVD segments demonstrate oppositional regression of the right superolateral regions paired with right inferolateral regression, with the L1-L2 IVD demonstrating a near oppositional response on the left side (5A). The L1-L2 shape change may be more characteristic of IVD changes with rotation, while the L2-L3 shape change (5B) may be more characteristic of shape changes more representative of side bend, suggesting limited rotation ROM available in more caudal segments. Range of disc shape change represents ±2 standard deviations.

For the L1-L2 IVDs, mode 3 (explained variance = 13.2%) demonstrated a comparable rotational deformation pattern (PC score difference: −6.28 [−11.03; −1.51], p = 0.01) as mode 4 of the primary analysis, with a similar left superolateral and right inferolateral expansion (1.3%), but with a more defined left inferolateral and right superolateral regression (−1.8%). Shapiro-Wilk test was significant for violation of normality in the change in shape for this mode (W = 0.65, p = 0.003). Paired permutation confirmed the significance of the shape change (Δ = −6.28 [−9.64; −2.92], p = 0.04). The shape deformation model is represented in Fig. 5A (values summarized in Table 3). Mode 4 (explained variance = 6.8%) in the L2-L3 IVDs demonstrated more of a unique response (PC score difference: −4.08 [−8.13; −0.04], p = 0.047), with a right inferolateral expansion (1.9%) and a right superolateral regression (−1.4%), representing more IVD displacement rather than rotation. The model is visualized in Fig. 5B (values summarized in Table 3). These patterns represent regions of the respective IVDs experiencing sustained compression and expansion as a result of the applied SRM.

## 4 Discussion

This proof-of-concept study mechanistically demonstrated a measurable, acute deformation of lumbar IVDs in five healthy, asymptomatic participants following a sustained unilateral SRM. As hypothesized, the primary analysis revealed direction-specific changes in IVD shape, further validated by excellent segmentation reliability and SSM metrics. This change is consistent with the theoretical expectation in accordance with the right-sided compression and left-sided traction of the spine in the left rotated position. Notably, the secondary analyses highlighted that significant shape changes were primarily observed in the smaller, cranial lumbar IVDs (L1-L2 and L2-L3), while the larger, caudal IVDs (L3-L4, L4-L5, and L5-S1) did not exhibit significant deformation. These findings suggest that the responsiveness of IVD morphology to therapeutic mobilizations is potentially modulated by anatomical and biomechanical factors, such as segmental mobility, forces acting on the individual segments, and IVD composition.

The observed differences between cranial and caudal IVDs are consistent with prior work demonstrating segment-specific deformation potential across the lumbar spine. Cranial discs are smaller, experience lower axial loading, and exhibit greater range of motion than caudal segments, which are structurally adapted for load-bearing and stability [27,28]. Anatomical features of the lower lumbar discs, including a thicker annulus fibrosus and higher collagen content, may further increase rigidity and resistance to deformation under load [29,30]. The absence of significant changes in the lower IVDs may also reflect limited statistical power in this small sample. Based on the largest observed effect sizes (d = 0.82–0.94 across lower segments), post-hoc power analyses suggest that approximately 14 participants would be required to achieve 80% power. Although limited sample size may have obscured subtler changes in the lower segments, the consistent responses observed in the upper lumbar spine suggest greater mechanical responsiveness to external mobilization. Clinically, this may indicate that SRM exerts a greater mechanical effect on upper lumbar segments, whereas responses in lower segments may be more constrained. This interpretation should be approached cautiously given the healthy sample, but it highlights the potential for segment-specific responsiveness to influence the effectiveness of manual interventions. Larger studies, particularly in symptomatic populations, are needed to confirm whether differential responses across lumbar levels have clinical significance.

The absence of significant deformation in the lower lumbar IVDs may reflect subtle differences in participant positioning, inter-individual anatomy, or biomechanical distinctions between cranial and caudal segments. The lack of consistent volumetric change across most IVDs suggests that the observed morphological differences were primarily shape-related rather than driven by fluid redistribution, consistent with prior cadaveric studies demonstrating direction-specific deformation of the nucleus pulposus and annulus fibrosus under mechanical loading [11,13]. Notably, a significant increase in L5-S1 IVD volume was observed, driven primarily by two participants who exhibited increases of 11.9% and 7.5% relative to baseline, substantially larger than the 2.9% average increase observed in the remainder of the sample. These two participants had relatively smaller L5–S1 discs compared with their L4-L5 levels, which may reflect asymptomatic degenerative changes and increased responsiveness to unloading by the time of the third acquisition [31]. Alternatively, the rotation mobilization may have produced a generalized increase in L5-S1 volume across the sample without a detectable direction-specific shape effect, as all participants demonstrated some increase in L5-S1 volume [32]. This may help explain the limited shape response in the most caudal segments, as changes in size without directional deformation would not be captured by the statistical shape model due to Procrustes scaling.

Taken together, the significant changes observed in the primary analysis, which included all lumbar IVD segments, suggest that SRM induces a consistent, direction-specific effect throughout the lumbar spine, albeit more pronounced in the upper lumbar IVDs. This response may be attributed to the hydrostatic nature of the IVD, where compressive forces on one side of the IVD cause deformation toward the area of least resistance [11]. This further supports the utility of SSM in detecting subtle morphological changes and highlights the importance of refining the study design to optimize clinical relevance and to better understand the mechanisms at play. Additionally, the variability in response across segments underscores the importance of segment-specific analysis when evaluating IVD behavior under mechanical loading.

Strengths of this study include the use of high-resolution MRI, SSM, and the practical in vivo application of a clinical SRM suspected to influence IVD behavior. The T1 VIBE sequence provided detailed visualization of soft tissue structures and enabled precise and repeatable segmentation performance. More specifically, with the significant shape changes detected representing a redistribution of disc volume between 1.3% and 2.5%, representing approximately 180 mm^3^ based on the average disc volume, or 200 voxels of redistributed disc material given the voxel size (0.82 mm^3^). The use of SSM offered a robust method with strong construct validity, allowing for the quantification of complex morphological changes not easily defined with uni-planar methods. Furthermore, the pragmatic design mimicked clinical practice methods, demonstrating that a common side-lying rotation mobilization position can produce a measurable response in healthy IVD shape.

The study also has notable limitations. The small sample size limits generalizability and may have reduced sensitivity to detect differences in more caudal IVD segments. Participants were generally of similar stature; however, Participant 5 was considerably taller than the remainder of the sample (1.90 m vs. 1.63–1.70 m), which may have influenced the magnitude of change in IVD volume and shape. While the application of SSM was a strength, the degree of smoothing required to generate meshes representative of baseline IVD variation may have reduced sensitivity to more nuanced shape changes. From a mechanistic perspective, although the practitioner had over 30 years of clinical experience and applied the mobilization as consistently as possible, the use of sensors such as strain gauges or pressure mats could further standardize applied rotational forces. As participants were asymptomatic, findings may not generalize to individuals with chronic LBP, and we cannot comment on the relationship between IVD shape changes and symptom response. Finally, the T1 VIBE sequence required nearly 11 min for acquisition, allowing time for deformation responses to dissipate; faster sequences may better preserve transient changes, although resolution and contrast must be balanced.

Future research should aim to address these limitations by recruiting larger samples, standardizing the applied forces, and including anthropometric measures as covariates if statistical power permits. Previous studies have suggested that therapeutic mobilizations can induce centralization of symptoms by repositioning displaced nucleus pulposus or reducing intradiscal pressure [33,34], which is consistent with our findings from a biomechanical perspective. Additionally, investigating dose-response relationships by modulating the force and time of mobilization could provide valuable insights into the biomechanics of IVD deformation with therapeutic approaches. Research on symptomatic patients with discogenic LBP would be particularly valuable, as it could clarify the mechanistic relationships between IVD morphology, symptom modification, and changes in force and pressure within the IVD and on surrounding anatomy.

In summary, we found that a commonly used side-lying SRM used for discogenic low back pain was sufficient to yield a measurable, direction-specific response in lumbar IVDs, particularly in the L1-L2 and L2-L3 segments, and importantly in the intended direction. Future research can better evaluate the mechanical behavior of the IVD by investigating the possibility of a dose-response relationship by modulating applied parameters, as well as establishing clinical relevance or minimally important difference for patients with discogenic low back pain. These suggestions would build on the present findings and further improve the understanding of the relationship between IVD mechanics, symptoms, and sensitization of surrounding tissues.

## Ethics approval

The study was approved by Western University's Health Science Research Board (Study ID: 108497).

## Author contributions

JP and DMW contributed to the conception and design of the study, and acquisition of data. JP, HFA, and DMW all contributed to the analysis and interpretation of the data. JP, HFA, and DMW all contributed to the drafting and revision of the article for important intellectual content. JP, HFA, and DMW all approved the final version of the manuscript for submission. As the corresponding author, HFA takes responsibility for the integrity of the work.

## Declaration of Generative AI and AI-assisted technologies in the writing process

Nothing to disclose.

## Funding

Funding for the study was provided by the Pain and Quality of Life Integrative Research Lab, Faculty of Health Sciences, Western University, London, ON, and the Canada First Research Excellence Fund BrainsCAN, and BrainsCAN Platform Grant.

## Conflicts of interest

HFA is the owner MSK ImageWorks, a company providing medical image processing services. MSK ImageWorks services were not used for this study. There are no other conflicts of interest to declare.
