# Supplementary material for: Statistical shape modeling of MRI-based morphological response of lumbar intervertebral discs to a unilateral side lying spinal rotation mobilization
Source: Osteoarthr Cartil Open. 2026 Mar 14;8(2):100783. doi: 10.1016/j.ocarto.2026.100783 (PMC13049509; doi:10.1016/j.ocarto.2026.100783)
Supplement: Multimedia component 1 [file mmc1.docx]

SUPPLEMENTARY MATERIALS

| **Supplementary Table 1.** Participant and group PC scores for the first 10 modes representing IVD shape changes in the primary analysis (combining all IVD segments). Significant changes are represented in bold. | | | | | | | | |
| --- | --- | --- | --- | --- | --- | --- | --- | --- |
| **Participant** | **1** | **2** | **3** | **4** | **5** | **Mean (95%CI MD)** | ***p*** | **EV (%)** |
| Mode 1 – Pre | -19.45 | 3.22 | -28.38 | -28.45 | 59.21 | -2.77 ± 36.98 |  |  |
| Mode 1 – Post | -22.34 | 7.06 | 1.18 | -24.26 | 66.07 | 5.54 ± 36.57 |  |  |
| Mode 1 – ∆ | -2.89 | 3.84 | 29.56 | 4.18 | 6.87 | 8.32 (-2.63; 19.26) | 0.14 | 33.1 |
| Mode 2 – Pre | -8.14 | 1.74 | 3.23 | -0.38 | 4.71 | 0.23 ± 5.04 |  |  |
| Mode 2 – Post | -7.60 | -0.98 | 0.29 | 0.89 | 5.09 | -0.46 ± 4.59 |  |  |
| Mode 2 – ∆ | 0.54 | -2.72 | -2.94 | 1.27 | 0.38 | -0.69 (-7.21; 5.82) | 0.84 | 15.5 |
| Mode 3 – Pre | 11.29 | 4.42 | -9.28 | 8.37 | -19.75 | -0.99 ± 13.12 |  |  |
| Mode 3 – Post | 10.50 | -1.62 | 3.80 | 12.36 | -15.15 | 1.98 ± 11.07 |  |  |
| Mode 3 – ∆ | -0.78 | -6.04 | 13.08 | 3.99 | 4.59 | 2.97 (-1.61; 7.55) | 0.21 | 9.0 |
| Mode 4 – Pre | -0.47 | -6.33 | -5.43 | 10.24 | 6.33 | 0.87 ± 7.26 |  |  |
| Mode 4 – Post | 0.59 | -5.76 | -7.94 | 7.96 | -3.52 | -1.73 ± 6.27 |  |  |
| Mode 4 – ∆ | 1.06 | 0.57 | -2.51 | -2.28 | -9.85 | **-2.60 (-4.79; -0.41)** | **0.02** | 6.8 |
| Mode 5 – Pre | 0.06 | 2.67 | -3.11 | 2.48 | -4.80 | -0.54 ± 3.34 |  |  |
| Mode 5 – Post | -1.49 | 5.26 | -3.68 | 3.23 | 2.07 | 1.08 ± 3.62 |  |  |
| Mode 5 – ∆ | -1.56 | 2.59 | -0.57 | 0.75 | 6.87 | 1.62 (-0.79; 4.02) | 0.19 | 5.0 |
| Mode 6 – Pre | -2.05 | 4.70 | -3.28 | 0.79 | 1.23 | 0.28 ± 3.11 |  |  |
| Mode 6 – Post | -4.14 | 2.23 | -2.60 | -0.26 | 1.99 | -0.56 ± 2.80 |  |  |
| Mode 6 – ∆ | -2.08 | -2.48 | 0.68 | -1.05 | 0.76 | -0.83 (-2.87; 1.21) | 0.43 | 3.7 |
| Mode 7 – Pre | -6.19 | 3.47 | 3.00 | 3.04 | -0.80 | 0.50 ± 4.12 |  |  |
| Mode 7 – Post | -6.00 | 0.92 | 2.23 | 1.63 | -3.82 | -1.01 ± 3.67 |  |  |
| Mode 7 – ∆ | 0.20 | -2.55 | -0.77 | -1.41 | -3.02 | **-1.51 (-2.65; -0.37)** | **0.009** | 3.0 |
| Mode 8 – Pre | 0.73 | 0.96 | -0.47 | -0.88 | -0.36 | -0.01 ± 0.80 |  |  |
| Mode 8 – Post | 1.23 | -0.72 | -0.54 | -0.07 | 0.15 | 0.01 ± 0.77 |  |  |
| Mode 8 – ∆ | 0.50 | -1.68 | -0.06 | 0.81 | 0.51 | 0.02 (-0.80; 0.83) | 0.97 | 2.3 |
| Mode 9 – Pre | 0.03 | 0.44 | 0.34 | 1.20 | -0.08 | 0.39 ± 0.50 |  |  |
| Mode 9 – Post | -0.78 | 0.33 | 0.46 | -2.25 | -1.61 | -0.77 ± 1.19 |  |  |
| Mode 9 – ∆ | -0.81 | -0.11 | 0.12 | -3.46 | -1.53 | -1.16 (-2.69; 0.37) | 0.14 | 1.7 |
| Mode 10 – Pre | -0.04 | -0.29 | 0.63 | -0.23 | 1.39 | 0.29 ± 0.71 |  |  |
| Mode 10 – Post | 1.08 | -0.45 | -0.34 | -0.52 | -2.71 | -0.59 ± 1.36 |  |  |
| Mode 10 – ∆ | 1.12 | -0.16 | -0.96 | -0.29 | -4.10 | -0.88 (-2.23; 0.47) | 0.20 | 1.4 |
| Group mean, 95% confidence intervals (95%CI), and mean differences (MD) presented are represented in principal component (PC) scores. PC scores represent each participant’s position on a given shape axis for a particular mode before (Pre) and after (Post) the rotation mobilization. Bolded results represent significant differences. *p* = *p*-value, EV = explained variance. | | | | | | | | |

| **Supplementary Table 2.** Participant and group PC scores for the first 10 modes representing L1-L2 IVD shape change in the secondary analysis. Significant changes are represented in bold. | | | | | | | | |
| --- | --- | --- | --- | --- | --- | --- | --- | --- |
| **Participant** | **1** | **2** | **3** | **4** | **5** | **Mean (95%CI MD)** | **p** | **EV (%)** |
| Mode 1 – Pre | -33.69 | 8.70 | -13.76 | -35.91 | 74.46 | -0.04 ± 45.37 |  |  |
| Mode 1 – Post | -44.82 | 14.13 | -14.02 | -44.00 | 89.11 | 0.08 ± 55.41 |  |  |
| Mode 1 – ∆ | -11.13 | 5.43 | -0.26 | -8.09 | 14.65 | 0.12 (-12.81; 13.05) | 0.99 | 41.7 |
| Mode 2 – Pre | 7.68 | -23.62 | 7.52 | -2.36 | 2.85 | -1.59 ± 12.99 |  |  |
| Mode 2 – Post | 8.57 | -18.63 | 6.01 | 3.57 | 16.33 | 3.17 ± 13.09 |  |  |
| Mode 2 – ∆ | 0.89 | 4.99 | -1.51 | 5.93 | 13.48 | 4.76 (-2.37; 11.88) | 0.19 | 19.9 |
| Mode 3 – Pre | -8.81 | -2.69 | 8.57 | 8.76 | 4.63 | 2.09 ± 7.66 |  |  |
| Mode 3 – Post | -13.29 | -7.37 | 3.45 | 4.76 | -8.47 | -4.18 ± 7.90 |  |  |
| Mode 3 – ∆ | -4.48 | -4.68 | -5.12 | -4.00 | -13.1 | **-6.28 (-11.03; -1.51)^W^**  ***-6.28 (-9.64; -2.92)*** | **0.01**  *0.04* | 13.2 |
| Mode 4 – Pre | 6.81 | -1.04 | -5.97 | 0.11 | 3.50 | 0.68 ± 4.82 |  |  |
| Mode 4 – Post | -0.44 | -0.35 | -9.36 | 6.78 | -3.46 | -1.37 ± 5.84 |  |  |
| Mode 4 – ∆ | -7.25 | 0.69 | -3.39 | 6.67 | -6.96 | -2.05 (-9.30; 5.21) | **0.59** | 7.3 |
| Mode 5 – Pre | 0.50 | 1.22 | 3.19 | -2.27 | 3.53 | 1.23 ± 2.34 |  |  |
| Mode 5 – Post | 8.36 | -2.63 | 0.16 | -10.46 | -7.81 | -2.48 ± 7.36 |  |  |
| Mode 5 – ∆ | 7.86 | -3.85 | -3.03 | -8.19 | -11.34 | -3.71 (-12.76; 5.34) | 0.42 | 4.6 |
| Mode 6 – Pre | -2.61 | -1.15 | -1.04 | -2.01 | 4.61 | -0.44 ± 2.90 |  |  |
| Mode 6 – Post | 2.84 | 0.74 | -1.88 | 7.95 | -5.23 | 0.88 ± 4.97 |  |  |
| Mode 6 – ∆ | 5.45 | 1.89 | -0.84 | 9.96 | -9.84 | 1.32 (-7.91; 10.56) | 0.79 | 3.3 |
| Mode 7 – Pre | -4.59 | 0.90 | -2.12 | 0.84 | -0.91 | -1.18 ± 2.29 |  |  |
| Mode 7 – Post | 5.17 | -1.31 | 2.84 | 3.01 | 2.03 | 2.35 ± 2.35 |  |  |
| Mode 7 – ∆ | 9.76 | -2.21 | 4.96 | 2.17 | 2.94 | 3.52 (-1.89; 8.93) | **0.20** | 2.6 |
| Mode 8 – Pre | 1.63 | 0.30 | -3.72 | 3.08 | 2.43 | 0.74 ± 2.70 |  |  |
| Mode 8 – Post | 0.17 | -4.68 | -1.06 | -0.97 | -0.91 | -1.49 ± 1.85 |  |  |
| Mode 8 – ∆ | -1.46 | -4.98 | 2.66 | -4.05 | -3.34 | -2.23 (-5.99; 1.52) | 0.24 | 2.0 |
| Mode 9 – Pre | -2.09 | -1.99 | -1.42 | 2.28 | 0.42 | -0.56 ± 1.88 |  |  |
| Mode 9 – Post | 4.27 | 2.10 | 0.96 | -1.99 | 0.25 | 1.12 ± 2.31 |  |  |
| Mode 9 – ∆ | 6.36 | 4.09 | 2.38 | -4.27 | -0.17 | 1.68 (-3.41; 6.76) | 0.52 | 1.3 |
| Mode 10 – Pre | 0.29 | 0.26 | 0.51 | -2.13 | 0.95 | -0.02 ± 1.21 |  |  |
| Mode 10 – Post | 0.99 | -0.48 | 1.39 | 0.30 | -1.96 | 0.05 ± 1.33 |  |  |
| Mode 10 – ∆ | 0.7 | -0.74 | 0.88 | 2.43 | -2.91 | 0.07 (-2.42; 2.56) | 0.96 | 1.3 |
| Group mean, 95% confidence intervals (95%CI), and mean differences (MD) presented are represented in principal component (PC) scores. PC scores represent each participant’s position on a given shape axis for a particular mode before (Pre) and after (Post) the rotation mobilization. Bolded results represent significant differences. A superscripted “W” (^W^) represents a positive Shapiro-Wilk test. Italicized results represent the adjusted values after permutation testing. *p* = *p*-value, EV = explained variance. | | | | | | | | |

| **Supplementary Table 3.** Participant and group PC scores for the first 10 modes representing L2-L3 IVD shape change in the secondary analysis. Significant changes are represented in bold. | | | | | | | | |
| --- | --- | --- | --- | --- | --- | --- | --- | --- |
| **Participant** | **1** | **2** | **3** | **4** | **5** | **Mean (95%CI MD)** | **p** | **EV (%)** |
| Mode 1 – Pre | -32.97 | 9.03 | -11.44 | -34.47 | 65.83 | -0.80 ± 41.29 |  |  |
| Mode 1 – Post | -32.16 | 8.59 | -8.87 | -30.04 | 70.34 | 1.56 ± 41.91 |  |  |
| Mode 1 – ∆ | 0.81 | -0.44 | 2.57 | 4.43 | 4.51 | 2.36 (-0.34; 5.05) | 0.08 | 32.0 |
| Mode 2 – Pre | 2.63 | -11.85 | -6.62 | 10.62 | 7.55 | 0.47 ± 9.49 |  |  |
| Mode 2 – Post | 0.54 | -14.10 | -3.20 | 6.29 | 5.79 | -0.94 ± 8.34 |  |  |
| Mode 2 – ∆ | -2.09 | -2.25 | 3.42 | -4.33 | -1.76 | -1.40 (-4.98; 2.17) | 0.45 | 22.4 |
| Mode 3 – Pre | 2.93 | 0.53 | -2.80 | 2.10 | -0.86 | 0.38 ± 2.30 |  |  |
| Mode 3 – Post | 0.95 | 13.32 | -19.34 | -0.53 | 1.79 | -0.76 ± 11.76 |  |  |
| Mode 3 – ∆ | -1.98 | 12.79 | -16.54 | -2.63 | 2.65 | -1.14 (-14.29; 12.01) | 0.87 | 17.0 |
| Mode 4 – Pre | -8.75 | 2.64 | 5.73 | 5.51 | 1.68 | 1.36 ± 5.92 |  |  |
| Mode 4 – Post | -10.24 | 1.00 | 2.89 | 0.34 | -7.60 | -2.72 ± 5.81 |  |  |
| Mode 4 – ∆ | -1.49 | -1.64 | -2.84 | -5.17 | -9.28 | **-4.08 (-8.13; -0.04)** | **0.04** | 6.8 |
| Mode 5 – Pre | -1.30 | -3.93 | 1.04 | 0.94 | -3.34 | -1.32 ± 2.32 |  |  |
| Mode 5 – Post | -4.39 | 7.71 | 2.67 | 1.45 | 5.74 | 2.64 ± 4.64 |  |  |
| Mode 5 – ∆ | -3.09 | 11.64 | 1.63 | 0.51 | 9.08 | 3.95 (-3.71; 11.62) | 0.32 | 6.1 |
| Mode 6 – Pre | -1.51 | 3.99 | -7.15 | 2.66 | -0.78 | -0.56 ± 4.34 |  |  |
| Mode 6 – Post | -1.13 | 2.77 | 1.39 | 2.62 | -0.05 | 1.12 ± 1.69 |  |  |
| Mode 6 – ∆ | 0.38 | -1.22 | 8.54 | -0.04 | 0.73 | 1.68 (-3.17; 6.53)^W^  *1.68 (-1.75; 5.11)* | 0.51  *0.59* | 3.4 |
| Mode 7 – Pre | -2.61 | 0.58 | 0.72 | 3.35 | -1.20 | 0.17 ± 2.25 |  |  |
| Mode 7 – Post | 1.13 | -2.64 | -1.06 | -1.46 | 2.35 | -0.34 ± 2.03 |  |  |
| Mode 7 – ∆ | 3.74 | -3.22 | -1.78 | -4.81 | 3.55 | -0.50 (-5.40; 4.39) | **0.85** | 2.7 |
| Mode 8 – Pre | -1.12 | 0.29 | -1.06 | 1.12 | 2.04 | 0.25 ± 1.37 |  |  |
| Mode 8 – Post | 0.76 | 1.52 | -0.70 | 0.20 | -4.32 | -0.51 ± 2.28 |  |  |
| Mode 8 – ∆ | 1.88 | 1.23 | 0.36 | -0.92 | -6.36 | -0.76 (-4.86; 3.34) | 0.72 | 2.7 |
| Mode 9 – Pre | 1.17 | -2.17 | -1.24 | -0.77 | 1.23 | -0.36 ± 1.51 |  |  |
| Mode 9 – Post | -0.86 | 2.05 | 4.12 | 0.27 | -2.02 | 0.71 ± 2.43 |  |  |
| Mode 9 – ∆ | -2.03 | 4.22 | 5.36 | 1.04 | -3.25 | 1.07 (-3.60; 5.73) | 0.67 | 2.0 |
| Mode 10 – Pre | 2.54 | 0.74 | 0.60 | 0.43 | 0.14 | 0.89 ± 0.95 |  |  |
| Mode 10 – Post | -6.39 | -1.59 | -1.91 | 1.22 | -0.23 | -1.78 ± 2.86 |  |  |
| Mode 10 – ∆ | -8.93 | -2.33 | -2.51 | 0.79 | -0.37 | -2.67 (-7.34; 2.00) | 0.27 | 1.4 |
| Group mean, 95% confidence intervals (95%CI), and mean differences (MD) presented are represented in principal component (PC) scores. PC scores represent each participant’s position on a given shape axis for a particular mode before (Pre) and after (Post) the rotation mobilization. A superscripted “W” (^W^) represents a positive Shapiro-Wilk test. Italicized results represent the adjusted values after permutation testing. *p* = *p*-value, EV = explained variance. | | | | | | | | |

| **Supplementary Table 4.** Participant and group PC scores for the first 10 modes representing L3-L4 IVD shape change in the secondary analysis. | | | | | | | | |
| --- | --- | --- | --- | --- | --- | --- | --- | --- |
| **Participant** | **1** | **2** | **3** | **4** | **5** | **Mean (95%CI MD)** | **p** | **EV (%)** |
| Mode 1 – Pre | -20.73 | -2.06 | -3.25 | -28.90 | 53.38 | -0.31 ± 32.13 |  |  |
| Mode 1 – Post | -25.33 | 5.00 | -2.18 | -28.73 | 54.36 | 0.63 ± 33.36 |  |  |
| Mode 1 – ∆ | -4.60 | 7.06 | 1.07 | 0.17 | 0.99 | 0.94 (-7.19; 9.06) | 0.83 | 37.9 |
| Mode 2 – Pre | 13.91 | -6.56 | 9.39 | -15.18 | -2.07 | -0.10 ± 11.83 |  |  |
| Mode 2 – Post | 13.02 | -6.34 | 3.63 | -10.00 | 0.71 | 0.20 ± 8.99 |  |  |
| Mode 2 – ∆ | -0.90 | 0.23 | -5.77 | 5.17 | 2.79 | 0.30 (-7.79; 8.40) | 0.95 | 21.1 |
| Mode 3 – Pre | 0.17 | -13.64 | -1.81 | 6.82 | 8.10 | -0.07 ± 8.68 |  |  |
| Mode 3 – Post | 5.56 | -7.73 | 4.84 | 3.05 | -5.01 | 0.14 ± 6.09 |  |  |
| Mode 3 – ∆ | 5.38 | 5.91 | 6.65 | -3.78 | -13.12 | 0.21 (-16.60; 17.01) | 0.98 | 10.8 |
| Mode 4 – Pre | 4.83 | 0.97 | -5.93 | 1.10 | 3.00 | 0.79 ± 4.08 |  |  |
| Mode 4 – Post | 5.74 | -2.72 | -13.89 | 1.20 | 1.72 | -1.59 ± 7.50 |  |  |
| Mode 4 – ∆ | 0.91 | -3.69 | -7.96 | 0.11 | -1.29 | -2.38 (-9.39; 4.62) | 0.51 | 8.2 |
| Mode 5 – Pre | 2.32 | 0.05 | -7.24 | -1.47 | -0.81 | -1.43 ± 3.55 |  |  |
| Mode 5 – Post | 3.85 | 8.38 | 4.43 | -2.39 | 0.00 | 2.86 ± 4.18 |  |  |
| Mode 5 – ∆ | 1.53 | 8.33 | 11.67 | -0.92 | 0.80 | 4.28 (-6.35; 14.91) | 0.44 | 6.0 |
| Mode 6 – Pre | -1.21 | 2.97 | 2.35 | 0.32 | 3.35 | 1.55 ± 1.94 |  |  |
| Mode 6 – Post | 1.59 | 0.26 | -2.39 | -5.42 | -9.59 | -3.11 ± 4.51 |  |  |
| Mode 6 – ∆ | 2.80 | -2.71 | -4.73 | -5.74 | -12.94 | -4.66 (-15.81; 6.48) | 0.42 | 4.7 |
| Mode 7 – Pre | 1.08 | 2.99 | -2.06 | -0.79 | 0.58 | 0.36 ± 1.91 |  |  |
| Mode 7 – Post | 0.03 | -1.31 | 2.00 | -2.65 | -1.67 | -0.72 ± 1.80 |  |  |
| Mode 7 – ∆ | -1.05 | -4.30 | 4.06 | -1.86 | -2.25 | -1.08 (-7.18; 5.02) | 0.74 | 2.6 |
| Mode 8 – Pre | -0.27 | -0.27 | 2.03 | 0.73 | -1.51 | 0.14 ± 1.32 |  |  |
| Mode 8 – Post | 0.23 | -0.83 | -3.42 | -0.61 | 3.21 | -0.28 ± 2.38 |  |  |
| Mode 8 – ∆ | 0.51 | -0.56 | -5.44 | -1.34 | 4.72 | -0.42 (-7.59; 6.74) | 0.92 | 1.7 |
| Mode 9 – Pre | -1.38 | -0.17 | -0.44 | 2.15 | -1.13 | -0.20 ± 1.40 |  |  |
| Mode 9 – Post | 3.08 | -0.90 | 2.00 | -4.80 | 2.58 | 0.39 ± 3.28 |  |  |
| Mode 9 – ∆ | 4.45 | -0.73 | 2.44 | -6.95 | 3.71 | 0.59 (-8.54; 9.71) | 0.91 | 1.7 |
| Mode 10 – Pre | 1.71 | -0.40 | 0.44 | 2.06 | -0.09 | 0.75 ± 1.09 |  |  |
| Mode 10 – Post | -2.75 | 1.03 | -1.53 | -4.27 | 0.04 | -1.50 ± 2.12 |  |  |
| Mode 10 – ∆ | -4.47 | 1.42 | -1.97 | -6.33 | 0.13 | -2.24 (-8.51; 4.02) | 0.49 | 1.7 |
| Group mean, 95% confidence intervals (95%CI), and mean differences (MD) presented are represented in principal component (PC) scores. PC scores represent each participant’s position on a given shape axis for a particular mode before (Pre) and after (Post) the rotation mobilization. *p* = *p*-value, EV = explained variance. | | | | | | | | |

| **Supplementary Table 5.** Participant and group PC scores for the first 10 modes representing L4-L5 IVD shape change in the secondary analysis. | | | | | | | | |
| --- | --- | --- | --- | --- | --- | --- | --- | --- |
| **Participant** | **1** | **2** | **3** | **4** | **5** | **Mean (95%CI MD)** | **p** | **EV (%)** |
| Mode 1 – Pre | -29.95 | -5.74 | 6.37 | -27.36 | 64.11 | 1.49 ± 38.14 |  |  |
| Mode 1 – Post | -26.62 | -0.78 | 0.72 | -26.73 | 64.06 | 2.13 ± 37.10 |  |  |
| Mode 1 – ∆ | 3.33 | 4.96 | -5.66 | 0.63 | -0.05 | 0.64 (-7.32; 8.60) | 0.88 | 55.4 |
| Mode 2 – Pre | 12.38 | -33.80 | -7.51 | 1.72 | 8.68 | -3.71 ± 18.45 |  |  |
| Mode 2 – Post | 23.09 | -22.26 | 3.61 | 1.20 | 1.40 | 1.41 ± 16.09 |  |  |
| Mode 2 – ∆ | 10.72 | 11.54 | 11.11 | -0.52 | -7.28 | 5.11 (-11.69; 21.92) | 0.56 | 21.8 |
| Mode 3 – Pre | -12.72 | -1.70 | 2.16 | 16.72 | 8.24 | 2.54 ± 11.00 |  |  |
| Mode 3 – Post | 0.16 | -2.42 | -16.59 | 11.71 | -9.63 | -3.35 ± 10.66 |  |  |
| Mode 3 – ∆ | 12.88 | -0.73 | -18.75 | -5.00 | -17.87 | -5.89 (-31.62; 19.83) | 0.67 | 7.5 |
| Mode 4 – Pre | 7.83 | -0.16 | 11.07 | 1.97 | 2.73 | 4.69 ± 4.62 |  |  |
| Mode 4 – Post | -4.80 | -1.92 | -1.76 | 4.34 | 1.52 | -0.52 ± 3.52 |  |  |
| Mode 4 – ∆ | -12.62 | -1.76 | -12.83 | 2.37 | -1.21 | -5.21 (-19.01; 8.59) | 0.47 | 4.1 |
| Mode 5 – Pre | 1.81 | 1.46 | 3.84 | -0.49 | -1.11 | 1.10 ± 1.97 |  |  |
| Mode 5 – Post | 8.28 | 2.08 | -7.37 | -3.29 | 4.63 | 0.87 ± 6.24 |  |  |
| Mode 5 – ∆ | 6.48 | 0.62 | -11.21 | -2.80 | 5.74 | -0.24 (-14.38; 13.91) | 0.98 | 3.1 |
| Mode 6 – Pre | 2.20 | 2.37 | 0.27 | -0.30 | 4.42 | 1.79 ± 1.88 |  |  |
| Mode 6 – Post | 1.69 | -0.19 | -0.84 | -8.30 | -9.22 | -3.37 ± 5.02 |  |  |
| Mode 6 – ∆ | -0.51 | -2.56 | -1.10 | -7.99 | -13.64 | -5.16 (-16.11; 5.79) | 0.36 | 2.0 |
| Mode 7 – Pre | -0.61 | 1.87 | -0.73 | -3.98 | 0.89 | -0.51 ± 2.22 |  |  |
| Mode 7 – Post | 4.13 | -1.24 | -2.42 | 3.42 | -1.67 | 0.44 ± 3.08 |  |  |
| Mode 7 – ∆ | 4.74 | -3.10 | -1.69 | 7.40 | -2.56 | 0.96 (-8.43; 10.34) | 0.85 | 1.7 |
| Mode 8 – Pre | 0.27 | -1.52 | 1.38 | 0.28 | 0.40 | 0.16 ± 1.05 |  |  |
| Mode 8 – Post | -0.17 | 4.45 | 1.65 | -0.72 | -1.31 | 0.78 ± 2.33 |  |  |
| Mode 8 – ∆ | 0.46 | -0.53 | -0.55 | 0.08 | -0.82 | 0.62 (-5.41; 6.65) | 0.85 | 1.4 |
| Mode 9 – Pre | -1.32 | 2.47 | -2.80 | -0.11 | 2.27 | -0.27 ± 0.53 |  |  |
| Mode 9 – Post | -1.79 | 3.00 | -2.25 | -0.20 | 3.09 | 0.10 ± 2.28 |  |  |
| Mode 9 – ∆ | 0.46 | -0.53 | -0.55 | 0.08 | -0.82 | 0.37 (-4.64; 5.39) | 0.89 | 0.7 |
| Mode 10 – Pre | -1.79 | 0.54 | 2.11 | 1.06 | -0.46 | 0.29 ± 1.49 |  |  |
| Mode 10 – Post | 2.34 | -0.94 | 4.75 | -1.18 | 0.55 | 1.10 ± 2.47 |  |  |
| Mode 10 – ∆ | 4.12 | -1.49 | 2.64 | -2.24 | 1.01 | 0.81 (-4.46; 6.08) | 0.78 | 0.7 |
| Group mean, 95% confidence intervals (95%CI), and mean differences (MD) presented are represented in principal component (PC) scores. PC scores represent each participant’s position on a given shape axis for a particular mode before (Pre) and after (Post) the rotation mobilization. *p* = *p*-value, EV = explained variance. | | | | | | | | |

| **Supplementary Table 6.** Participant and group PC scores for the first 10 modes representing L5-S1 IVD shape change in the secondary analysis. | | | | | | | | |
| --- | --- | --- | --- | --- | --- | --- | --- | --- |
| Participant | 1 | 2 | 3 | 4 | 5 | Mean (95%CI MD) | **p** | **EV (%)** |
| Mode 1 – Pre | -20.82 | -9.61 | 3.17 | -36.78 | 57.54 | -1.29 ± 36.02 |  |  |
| Mode 1 – Post | -18.26 | -7.81 | 6.59 | -24.48 | 56.94 | 2.60 ± 32.58 |  |  |
| Mode 1 – ∆ | 2.56 | 1.79 | 3.42 | 12.31 | -0.60 | 3.89 (-5.78; 13.57) | 0.44 | 49.4 |
| Mode 2 – Pre | -9.40 | 23.64 | 12.02 | -16.16 | -15.54 | 1.09 ± 17.95 |  |  |
| Mode 2 – Post | -12.80 | 17.11 | 15.37 | -10.58 | 1.78 | 2.18 ± 14.00 |  |  |
| Mode 2 – ∆ | 3.40 | 6.52 | -3.35 | -5.57 | -17.32 | -3.26 (-21.41; 14.89) | 0.74 | 18.8 |
| Mode 3 – Pre | 11.94 | -9.18 | 9.37 | -11.74 | -3.93 | 0.71 ± 10.79 |  |  |
| Mode 3 – Post | 14.45 | -4.09 | 6.60 | -7.01 | -2.88 | -1.41± 8.89 |  |  |
| Mode 3 – ∆ | -2.51 | -5.09 | 2.77 | -4.73 | -1.05 | -2.12 (-8.38; 4.14) | 0.53 | 9.1 |
| Mode 4 – Pre | 7.34 | 7.14 | -9.64 | -4.93 | -0.11 | -0.04 ±7.45 |  |  |
| Mode 4 – Post | 5.46 | 0.03 | -6.73 | -2.31 | 3.95 | -0.08 ± 4.90 |  |  |
| Mode 4 – ∆ | 1.88 | 7.12 | -2.90 | -2.61 | -4.06 | -0.11 (-9.19; 8.96) | 0.98 | 5.9 |
| Mode 5 – Pre | 2.75 | 0.81 | 1.59 | 2.90 | 2.11 | 2.03 ± 0.86 |  |  |
| Mode 5 – Post | -2.21 | 1.85 | -2.18 | -13.70 | -4.11 | -4.07 ± 5.81 |  |  |
| Mode 5 – ∆ | 4.96 | -1.04 | 3.77 | 16.60 | 6.22 | 6.10 (-6.60; 18.80) | 0.35 | 4.1 |
| Mode 6 – Pre | -2.06 | 2.47 | -1.03 | 1.19 | 2.53 | 0.62 ± 2.08 |  |  |
| Mode 6 – Post | 2.80 | -6.72 | 7.64 | -3.27 | -6.64 | -1.24 ± 6.30 |  |  |
| Mode 6 – ∆ | -4.86 | 9.19 | -8.67 | 4.46 | 9.17 | 1.86 (-14.25; 17.96) | 0.83 | 3.4 |
| Mode 7 – Pre | 0.94 | -0.50 | -0.08 | 0.48 | 0.75 | 0.32 ± 0.60 |  |  |
| Mode 7 – Post | -2.02 | 5.51 | -4.15 | -1.41 | -1.09 | -0.63 ± 3.63 |  |  |
| Mode 7 – ∆ | 2.96 | -6.01 | 4.07 | 1.89 | 1.83 | -0.95 (-6.88; 8.78)^W^  *-0.95 (-4.46; 2.56)* | 0.82  *0.70* | 2.5 |
| Mode 8 – Pre | 1.19 | 2.20 | 0.42 | 2.03 | -0.99 | 1.31 ± 0.97 |  |  |
| Mode 8 – Post | -1.36 | -6.87 | -1.17 | -3.68 | 3.37 | -1.94 ± 3.76 |  |  |
| Mode 8 – ∆ | 2.55 | 9.07 | 1.59 | 5.71 | -4.36 | -2.91 (-6.91; 12.73) | 0.57 | 1.6 |
| Mode 9 – Pre | 0.60 | -0.13 | 0.48 | -0.06 | -1.67 | -0.16 ± 0.91 |  |  |
| Mode 9 – Post | -2.08 | -4.11 | 1.40 | 3.18 | 3.16 | 0.31 ± 3.27 |  |  |
| Mode 9 – ∆ | 2.68 | 3.98 | -0.93 | -3.24 | -4.83 | -0.47 (-7.84; 6.91) | 0.91 | 1.3 |
| Mode 10 – Pre | -0.29 | -0.25 | 0.90 | 1.30 | -1.58 | 0.02 ± 1.13 |  |  |
| Mode 10 – Post | 0.02 | -0.50 | -1.32 | -2.10 | 3.74 | -0.03 ± 2.26 |  |  |
| Mode 10 – ∆ | -0.31 | 0.25 | 2.22 | 3.40 | -5.32 | 0.05 (-6.52; 6.62) | 0.98 | 1.3 |
| Group mean, 95% confidence intervals (95%CI), and mean differences (MD) presented are represented in principal component (PC) scores. PC scores represent each participant’s position on a given shape axis for a particular mode before (Pre) and after (Post) the rotation mobilization. A superscripted “W” (^W^) represents a positive Shapiro-Wilk test. Italicized results represent the adjusted values after permutation testing. *p* = *p*-value, EV = explained variance. | | | | | | | | |
